# Associations of feeding and delivery modes with the risk of otitis media with effusion in children: A cross-sectional study

**DOI:** 10.1097/MD.0000000000044692

**Published:** 2026-05-12

**Authors:** Yan Wang, Yuanjia Hu, Hong Lin, Yunyun Pan, Zhaoyi Zhou, Chenyu Zhu, Jiacheng Wang, Yaowen Wang

**Affiliations:** a Ear, Nose and Throat Department, The First Affiliated Hospital of Ningbo University, Ningbo, China; b Department of Voice, Zhongshan Hospital of Xiamen University, School of Medicine, Xiamen University, Xiamen, China; c Department of Infectious Diseases, The First Affiliated Hospital of Ningbo University, Ningbo, China; d School of Medical Technology and Information Engineering, Zhejiang Chinese Medical University, Hangzhou, China.

**Keywords:** breastfeeding, cesarean section, formula feeding, infection, natural birth, OME

## Abstract

Otitis media with effusion (OME) is predominantly observed in children under 6 years of age and can lead to hearing loss or even severe complications, impacting speech, and cognitive development. While the benefits of breastfeeding and natural birth in enhancing infant immunity and cognitive abilities are globally recognized, their effects on OME have been scarcely reported. This study investigates the relationship between feeding methods and delivery modes with OME in children, aiming to identify potential associative factors that may inform health education and early risk awareness, where medically feasible. A total of 81 children diagnosed with OME, aged ≤6 years, from May 2022 to May 2023, were selected as the experimental group. Another 81 children without a history of OME served as the control group. A questionnaire was used to inquire about the feeding methods and delivery modes at birth, as reported by the guardians. Statistical analysis was performed using SPSS 20.0 software. Formula feeding and cesarean section are positively correlated with the incidence of OME (*r* > 0, *P* < .001), indicating that these factors contribute to an increased prevalence of the condition. A combined analysis of feeding and birth modes reveals that the incidence of OME is significantly higher in the group where formula feeding is combined with cesarean section, suggesting that these effects are additive. Compared with formula feeding, breastfeeding significantly reduces the risk of OME infections; similarly, natural birth is more effective than cesarean section in lowering the incidence of OME. When both feeding and delivery modes are considered together, breastfeeding can mitigate the risk of OME in children born via cesarean section, while natural birth can decrease the risk associated with formula feeding children. Therefore, the methods of feeding and delivery modes are closely linked to the early incidence of OME in children. Where medically appropriate, continued promotion of breastfeeding and support for vaginal delivery may be beneficial in reducing risk.

## 1. Introduction

Otitis media with effusion (OME) is a common pediatric condition, predominantly seen in children under the age of 6.^[1]^ Characterized by fluid accumulation in the middle ear and associated hearing loss, it is clinically referred to as serous or nonsuppurative otitis media.^[2]^ Despite its self-limiting nature, the fluid can interfere with auditory function, potentially leading to bacterial otitis media and more severe complications such as tympanic membrane atelectasis, adhesive otitis media, and cholesteatoma.^[3]^ These complications increase the risk of hearing loss in children, which can significantly impact their speech and cognitive development, leading to serious adverse outcomes.

Breastfeeding is recognized as a protective factor against childhood diseases. The physiological process of establishing a healthy gut microbiota in infants aids in the maturation of the immune system and helps prevent allergies. It has been shown to reduce the incidence of necrotizing enterocolitis in newborns,^[4]^ diminish the occurrence of developmental delays at age 1,^[5]^ enhance cognitive development in children,^[6]^ and improve executive functions.^[7]^

However, current research has not revealed the impact of breastfeeding and modes of delivery on the incidence of otitis media in children. Therefore, this study surveys the caregivers of 162 children to analyze the associations between feeding and delivery modes and the risk of OME. While recognizing that these factors are often determined by clinical conditions, the findings may provide insight into early modifiable exposures in appropriate cases.

## 2. Methods

### 2.1. Participants

Eighty-one children aged 6 years or younger were selected as the experimental group, while 81 children without a history of the condition comprised the control group. Caregivers were surveyed to evaluate the impact of feeding and delivery modes on reducing the risk of OME in children. We confirm that informed consent was obtained from all participants and/or their legal guardians, and the research was conducted in accordance with the Declaration of Helsinki.

### 2.2. Survey

The survey covered a range of topics, including maternal age at childbirth, maternal infections during pregnancy, high-risk factors during delivery, child’s age, gender, birth weight, prematurity, feeding and delivery methods, as detailed in Table 1.

**Table 1:** Questionnaire on basic information.

Survey items	Responses
1. Child’s age	__________
2. Child’s gender	□Male □Female
3. Maternal age at childbirth	__________
4. High-risk factors during pregnancy	☐No ☐Yes: __________
5. Was the delivery process smooth for mother?	☐Yes ☐No: reason __________
6. Was the birth full-term? (≥37 weeks)	☐Yes ☐No
7. Baby’s weight at birth	__________
8. Were there any high-risk factors at birth	☐No ☐Yes: __________
9. Feeding method	☐Breastfeeding ☐Formula
10. Delivery method	☐Natural birth ☐Cesarean section

Otolaryngology technicians administered questionnaires to the caregivers after obtaining informed consent. The diagnosis of OME was based on clinical criteria and confirmed by professional medical diagnosis. All questionnaires had to be fully completed by the caregivers to be considered valid.

### 2.3. Inclusion and exclusion criteria

Inclusion criteria: age ≤6 years; tympanometric curve types B or C; visible changes in the color or retraction of the tympanic membrane or prominence of the malleus under otoscopy; presence of an air-fluid level or bubbles on tympanic membrane observed during otoscopic examination, meeting the clinical diagnostic criteria for OME.

Exclusion criteria: maternal age over 35 years; maternal infection during pregnancy; premature infants (<37 weeks); children born with high-risk factors; age >6 years; incomplete questionnaire responses.

### 2.4. Statistical analysis

Data were analyzed using SPSS (version 20.0; IBM Corp., Armonk). The chi-square test was used to compare categorical variables between groups. The associations between feeding methods, delivery modes, and OME were analyzed using binary logistic regression. A *P*-value <.05 was considered statistically significant.

## 3. Results

### 3.1. Statistical findings on the correlation between OME, feeding methods, and birth modes

The results indicate a significant correlation between the incidence of OME and both feeding and delivery methods (Table 2).

**Table 2:** Correlation results for OME, feeding methods, and delivery modes.

Variable	*β*	SE	Wald *χ*²	OR	95% CI (lower)	95% CI (upper)	*P*
OME–feeding methods	0.927	0.419	4.895	2.527	1.112	5.744	.027
OME–delivery modes	1.162	0.484	5.764	3.196	1.238	8.254	.017

OME = otitis media with effusion.

### 3.2. Statistical outcome for feeding and birth methods between groups

As depicted in Table 3, in the control group, 61 instances (75.31%) of breastfeeding and 20 instances (24.69%) of formula feeding were recorded. In contrast, the experimental group showed 27 instances (33.33%) of breastfeeding and 54 instances (66.67%) of formula feeding. Chi-square test results indicated a significant difference in feeding methods between the groups. For delivery methods, the control group had 28 cesarean sections (34.57%) and 53 natural births (65.43%). The experimental group consisted of 49 cesarean sections (60.49%) and 32 natural births (39.51%). Chi-square test results also showed significant differences in birth methods between the groups.

**Table 3:** Differences in feeding and delivery modes between groups.

Group	Breastfeeding	Formula feeding	Cesarean section	Natural birth
Control group	61 (75.31%)	20 (24.69%)	28 (34.57%)	53 (65.43%)
OME group	27 (33.33%)	54 (66.67%)	49 (60.49%)	32 (39.51%)
Chi-square value	27.091		9.901	
*P*-value	<.001		<.01	

OME = otitis media with effusion.

### 3.3. Statistical results of feeding and birth methods in the OME group

Among 81 children with OME, 54 (66.67%) were formula-fed and 27 (33.33%) were breastfed, with formula feeding showing a significantly higher proportion. Cesarean sections were more prevalent at 49 cases (60.49%) compared with 32 natural births (39.51%), with a statistically significant difference observed (Table 4).

**Table 4:** Comparison of feeding and birth methods among OME group.

OME group	Breastfeeding	Formula feeding	Cesarean section	Natural birth
	27 (33.33%)	54 (66.67%)	49 (60.49%)	32 (39.51%)
Chi-square value	16.691		6.321	
*P*-value	<.001		<.01	

OME = otitis media with effusion.

### 3.4. Statistical comparison of feeding and birth method combinations in OME group

Table 5 indicated that group A, with formula feeding and cesarean section, had a higher incidence of OME, showing statistically significant differences. Groups B and D showed no significant differences with near-equal ratios. Group C showed a significantly higher incidence in the formula feeding and cesarean section. Group E also showed a higher incidence in the formula feeding and cesarean section. Group F showed no significant difference.

**Table 5:** Comparison of feeding and birth method combinations in OME group.

Group	Combination	Percentage	Chi-square value	*P*-value
A	Formula + cesarean vs formula + natural	66.67% vs 33.33%	10.704	.001
B	Breast + cesarean vs breast + natural	48.15% vs 51.85%	0.008	.782
C	Formula + cesarean vs breast + cesarean	73.47% vs 26.53%	19.755	<.001
D	Formula + natural vs breast + natural	56.25% vs 43.75%	0.563	.453
E	Formula + cesarean vs breast + natural	72.00% vs 28.00%	17.640	<.001
F	Formula + natural vs breast + cesarean	58.06% vs 41.94%	1.032	.310

## 4. Discussion

OME is a prevalent condition in pediatric otolaryngology, with consistently high incidence and prevalence rates.^[8]^ Statistics reveal that the occurrence rate of OME in Chinese children ranges from 5.2% to 21.6%, while it varies between 6% and 20% internationally.^[9]^ The anatomical structure of children’s eustachian tubes – short, flat, and wide – facilitates the accumulation of nasopharyngeal inflammation, often leading to tube narrowing or obstruction, a common cause of OME in children. Additionally, since OME typically does not produce pronounced ear pain and children may not clearly articulate their discomfort, diagnosis often occurs only after significant hearing impairment is noticed by parents, resulting in delays in both diagnosis and treatment.

The etiology of OME is multifactorial; studies have demonstrated a significant association between the condition and feeding methods.^[10,11]^ Our analysis on the correlation between OME and both feeding and birth methods has identified significant relationships, with feeding methods showing a notable correlation (*r* = 0.421, *P* < .001) and birth methods also demonstrating a significant association (*r* = 0.260, *P* < .001).

Breast milk, unlike infant formula, is a bioactive substance replete with antibacterial, anti-inflammatory, and immunomodulatory agents that compensate for the physiological immaturity of an infant’s immune system.^[12,13]^ It provides secretory IgA and activated T-cell antibodies against various gastrointestinal and respiratory pathogens prevalent in the mother–infant environment. Additionally, breast milk supplies leukocytes and lysosomes that offer nonspecific defense against bacterial pathogens, significantly reducing the risk of respiratory, gastrointestinal, and ear infections, among others.^[12]^ The act of breastfeeding not only fosters a unique physiological interaction involving crucial bacterial and hormonal exchanges between mother and child, but it also creates a beneficial pressure gradient through vigorous sucking and swallowing motions. This pressure is essential for ventilating the eustachian tubes.^[14]^ When oxytocin stimulates the release of milk through myoepithelial cells in the ducts, a portion of the milk is repeatedly ejected, creating active pressure within the gland without accumulating negative pressure. Conversely, when infants use a bottle, they tend to prefer milk flowing from the bottle due to gravity or minor pressure from the oral cavity, resulting in negative intracavitary pressure that can transfer from the bottle to the middle ear, potentially causing eustachian tube dysfunction. This mechanism makes bottle-fed infants particularly susceptible to OME, especially when using nonventilated or poorly ventilated bottles.^[12,15]^

A systematic review in *The Lancet* regarding the outcomes of breastfeeding on maternal and child health found numerous health benefits for both, and the findings confirm the crucial role of breastfeeding in preventing common pediatric infections, including OME.^[16]^ Our study surveyed feeding methods among 81 children with OME and 81 without, finding a significantly higher rate of formula feeding (66.67%) in the experimental group compared to the control group (24.69%). The comparison within the experimental group also revealed a higher rate of formula feeding than breastfeeding (66.67% vs 33.33%), indicating a statistically significant difference. Among children with OME who underwent cesarean delivery, those fed formula (36 instances) outnumbered those breastfed (13 instances), confirming formula feeding as a significant risk factor for this condition (chi-square value = 10.704, *P* < .001). The study did not specifically research the correlation between exclusive breastfeeding duration and the incidence of OME, but the benefits of exclusive breastfeeding in reducing the risk of this infection were also evident, highlighting the protective effect of breastfeeding against the infection rate of OME.

Since 2000, the global cesarean section rate has increased annually by 3.7%, reaching 21.1% by 2015.^[17]^ This exceeds the World Health Organization’s threshold of 15%,^[18]^ with China’s rate notably higher at 46.5%. Cesarean deliveries pose significant health risks to both mothers and infants. Research indicates that the incidence of secretory otitis media (SOM) is associated with the method of delivery.^[19]^ This study found that the cesarean rate in the experimental group was 60.49%, substantially higher than the 39.51% in the control group. The proportion of cesarean deliveries was significantly greater than that of vaginal births. Additionally, among infants fed formula who had SOM, those born via cesarean showed a markedly higher rate, with significant differences (chi-square value = 10.704, *P* < .001), suggesting that vaginal births may reduce the risk of SOM more effectively than cesarean births.

Potential causes of differing impacts of birth modes on the incidence of SOM include impact on microbial colonization in early life: natural childbirth leads to the initial colonization of the infant’s intestines by maternal bacteria, a process vital for the development and maintenance of a healthy immune system.^[20–22]^ In contrast, infants delivered by cesarean section are primarily exposed to microbes found on the mother’s skin,^[23]^ altering their microbial colonization patterns.^[24]^ This difference soon after birth results in a distinct microbiome compared to natural delivered children,^[25–28]^ potentially affecting the natural development of the immune system and increasing the risk of SOM. Eustachian tube structure: the occurrence of SOM in children is closely related to eustachian tube dysfunction.^[29]^ Studies have identified a substance within the eustachian tube that reduces surface tension and helps keep it open, maintaining pressure equilibrium between the middle ear and the exterior.^[30–32]^ The ventilation function of the eustachian tube in cesarean-delivered infants may be compromised due to the lack of compressive forces experienced during vaginal delivery, making them more susceptible to SOM. Persistent Eustachian tube dysfunction may lead to chronic middle ear conditions such as atelectatic otitis and retraction pocket formation, which can progress to acquired cholesteatoma. Cholesteatoma often requires tympanoplasty and may result in permanent conductive hearing loss. In severe cases, osseointegrated bone-conduction hearing devices may be needed, which can impact aesthetics and quality of life.^[33]^ These risks highlight the importance of early prevention through modifiable factors such as feeding and delivery modes. Cesarean delivery and neonatal respiratory diseases: cesarean delivery is a recognized factor contributing to neonatal respiratory conditions, particularly transient tachypnea of the newborn and respiratory distress syndrome.^[34–37]^ Additionally, conditions such as allergic diseases, recurrent upper respiratory infections, and frequent nasal discharge are risk factors for SOM in children.^[38]^ Modern bacteriology and virology suggest that 1 cause of SOM is bacteria and viruses from the upper respiratory tract infecting the middle ear via the eustachian tube.^[39]^ The resulting antigen–antibody complexes can exacerbate damage to the mucosal lining of the middle ear, leading to dysfunction of the mucociliary system and local immune responses, altering the physiological function of the nose–pharynx–eustachian tube–middle ear system, and increasing the risk of SOM due to the development of negative pressure within the middle ear.

When assessing the impact of feeding methods and birth methods on SOM, we discovered that the combination of formula feeding and cesarean delivery presents the highest incidence rate of infection, statistically significant compared with other combinations. The combination of breastfeeding with cesarean delivery mitigates the impact associated with cesarean births, resulting in no statistically significant difference in the incidence rates of SOM across different birth methods within the breastfeeding group (*P* = .782). The combination of vaginal birth and formula feeding reduces the risks associated with formula feeding, showing no statistically significant difference in the incidence rates of SOM across different feeding methods within the vaginal birth group (*P* = .453). Moreover, the comparison between the incidence rates of SOM in formula-fed vaginal births and breastfed cesarean births also showed no statistically significant difference (*P* = .310).

This study has several limitations. First, the relatively small sample size and single-center design may reduce the statistical power and generalizability of the results. Second, as an observational study, causal relationships cannot be established, and random assignment to different feeding or delivery methods is not ethically feasible. Third, data on breastfeeding duration were not collected, limiting assessment of potential dose–response or long-term effects. Additionally, factors such as labor duration and other perinatal variables were not included. Future studies should involve larger, multicenter cohorts with more detailed data collection and prospective designs to clarify the role of early life exposures in the development of OME.

## 5. Conclusion

In summary, feeding methods and delivery modes are associated with the incidence of SOM. Formula feeding and cesarean section both increase the risk of infection, and these effects can be cumulative. Conversely, breastfeeding and natural birth can reduce the risk of this infection. Specifically, breastfeeding diminishes the risk of SOM in cesarean-born infants, while natural delivery can decrease the risk in formula-fed infants. Moving forward, we should advocate for enhanced breastfeeding promotion and encourage natural births as strategies to potentially reduce the incidence of SOM. Additionally, advocating for routine annual ear and hearing examinations for children can facilitate early detection and treatment of SOM, thereby averting potential hearing loss and complications.

## Author contributions

**Conceptualization**: Yan Wang.

**Data curation**: Yan Wang, Hong Lin, Chenyu Zhu, Jiacheng Wang.

**Formal analysis**: Yuanjia Hu, Yunyun Pan.

**Investigation**: Zhaoyi Zhou, Chenyu Zhu.

**Methodology**: Yan Wang, Jiacheng Wang.

**Writing – review & editing**: Yan Wang, Yuanjia Hu, Yaowen Wang.

**Writing – original draft**: Yan Wang, Yuanjia Hu.

## Supplementary Material
