# Supplementary material for: Associations of feeding and delivery modes with the risk of otitis media with effusion in children: A cross-sectional study
Source: Medicine (Baltimore). 2026 May 12;104(49):e44692. doi: 10.1097/MD.0000000000044692 (PMC12688935; doi:10.1097/MD.0000000000044692)
Supplement: Supplementary file 1 [file medi-104-e44692-s001.pdf]

Multimedia Appendix 1. Informed consent form template

知情同意书

(版本号:V1.4, 版本日期：2022 年 01 月 20 日)

尊敬的患者：

医生已经确诊您患有听力损失。我们将邀请您参加一项“喂养方式及生产方式与儿童分泌性中耳炎的相关性研究”的临床研究,在您决定是否参加这项研究之前，请尽可能仔细阅读以下内容。它可以帮助您了解该项研究以及为何要进行这项研究，研究的程序和期限，参加研究后可能给您带来的益处、风险和不便。

一、　研究背景和研究目的

儿童分泌性中耳炎(otitis media with effusion，OME）是一种常见的儿童疾病，大多数儿童发生于6周岁以内，是以中耳积液及听力下降为主要特征的中耳非化脓性疾病，临床上又称为渗出性中耳炎、非化脓性中耳炎。中耳炎的病因学是多因素的，个体因素如遗传、解剖和免疫特征等，局部因素如婴儿的保育护理，经济及环境因素如贫穷、营养不良以及群居等，会增加病原体传播的风险。而中耳的负压被认为是浆液性中耳炎的前兆，积液会干扰听力，导致幼儿的语言和认知发育下降，同时负压也可能导致细菌性中耳炎和更严重的并发症如鼓膜闭锁不张、粘连性中耳炎和胆脂瘤等，因此预防儿童OME十分重要。有研究表明，分泌性中耳炎的发生率与喂养方式和生产方式有关，本研究选取2020年5月～2021年9月确诊为OME的≤6岁的儿童81例为研究对象，对其监护人进行问卷调查，分析喂养方式或生产方式与儿童OME的相关性，为儿童OME有针对性的预防提供理论依据。

二、　具体研究时间

研究时间：2022.02-2022.03。

三、　如果参加研究您需要做什么？

本研究为横断面研究。如果您符合纳入标准并自愿参加研究，需要签署书面的知情同意书，在宁波市第一医院耳鼻喉科门诊，填写基本信息表和问卷调查。完成该问卷大约需要 5 分钟。

如果在研究期间您出现任何不适，或病情发生新的变化或任何意外情况，包括在其它医疗机构住院、伤残等，不管是否与研究有关，务必及时通知您的医生，以便对此作出判断并给予适当的医疗处理或建议，保障您的安全。

四、参加本研究可能给您带来的受益

本问卷为作为课题研究的调查工具，通过参加本研究，您将对中耳炎的防治有更深入的了，同时您的研究数据对于今后中耳炎领域的研究也将非常重要。

您的负责医生将回答您在整个过程中遇到的任何问题，为您提供及时的帮助和指导，并确保试验的顺利进行。

五、参加研究可能的不良反应、风险以及风险防范措施

参与本项目不涉及任何常规诊疗之外的药物或医疗器械干预措施，不存在发生常规诊疗之外的不良反应的风险。且对您个人身份信息进行保密，避免可能披露的风险。

六、有关费用说明

本研究项目不收取常规诊疗之外的任何额外费用。

七、您个人信息的保密

您的医疗记录（研究病历/CRF、化验单等）将完整地保存在您所就诊的医院。研究者、伦理委员会和药品监督管理部门将被允许查阅您的医疗记录。任何有关本项研究结果的公开报告将不会披露您的个人身份。我们将在法律允许的范围内，尽一切努力保护您个人医疗资料的隐私。

八、可以自愿选择参加研究和中途退出研究

是否参加研究完全取决于您的意愿。您可以拒绝参加此项研究，或在研究过程中的任何时间退出本研究，这都不会影响您和医生间的关系，都不会影响对您的医疗或有其他方面利益的损失。

出于对您的最大利益考虑，医生或研究者可能会在研究过程中中止您继续参加本项研究。

九、伦理委员会

本研究已经得到宁波市第一医院医学伦理委员会审核，并获得了批准。有关伦理和权益事宜可联系宁波市第一医院医学伦理委员会办公室，电话：0574-87085233。

十、现在该做什么？

是否参加本项研究由您自己（和您的家人）决定。

在您做出参加研究的决定前，请尽可能向您的医生询问有关问题。

感谢您阅读以上材料。如果您决定参加本项研究，请告诉您的医生，他/她会为您安排一切有关研究的事务。请您保留这份资料。

———————————————————————————————————————

我确认已阅读并理解了本研究的知情同意书，自愿接受本研究中的治疗方法，并同意将我的医疗数据用于本研究的发表。

患者签名：______________ 联系方式：______________日期：____年__月__日______

代理人签名：（如需要）______________；与受试者关系________________

联系方式 __________________日期：____年__月__日__；

见证人：（如需要）：____________联系方式：____________日期：____年__月__日____

我确认已向患者解释了本研究的详细情况，包括其权力以及可能的受益和风险，并给其一份签署过的知情同意书副本。

研究者签名：_______________ 日期：日期：____年__月__日______

联系方式：（手机）__________________
